# Normative Beliefs and Aggression: The Mediating Roles of Empathy and Anger

**DOI:** 10.1007/s10578-023-01558-1

**Published:** 2023-06-22

**Authors:** Cara S Swit, Seth C Harty

**Affiliations:** 1 Faculty of Health, University of Canterbury, Christchurch, 8041 New Zealand; 2 Faculty of Science, University of Canterbury, Christchurch, New Zealand

**Keywords:** Relational aggression, Physical aggression, Proactive aggression, Reactive aggression, Early childhood, Empathy, Anger

## Abstract

This study examined a two-mediator model with both empathy and anger as mediators in the association between children’s normative beliefs about aggression and forms (relational and physical) and functions (reactive and proactive) of aggressive behavior. Ninety-eight children (54% males, *M*_*age*_=46.21months, *SD* = 8.84months) reported their approval of relationally and physically aggressive behaviors depicted in iconic (animation) and enactive (toy figurines) hypothetical scenarios. Children’s aggression, empathy and anger were measured using teacher reports. No main effects of normative beliefs about aggression on the corresponding aggressive behavior were found. Normative beliefs about aggression were negatively associated with empathy and empathy was significantly associated with relational aggression, suggesting that developing social emotional processes mediate the relation between social cognitions and aggression. Anger was associated with aggression, but not normative beliefs about aggression. The findings provide support for the distinction between subtypes of aggressive behavior in young children and the developing social-cognitive and affective processes that influence these behaviors.

**Supplementary Information:**

The online version contains supplementary material available at 10.1007/s10578-023-01558-1.

## Introduction

Forms of aggression are common and relatively typical during early childhood. While most children go on to develop socio-emotional and cognitive skills that mark a rapid decline in aggression, some children’s use of aggression continues to persist. Children who are persistently aggressive during early childhood are at greater risk of internalising consequences (e.g. clinical and subclinical depression and anxiety) and externalizing problems (e.g. peer victimization and delinquency) [1–4]. Over the years, researchers have emphasised the importance of early childhood in the onset of persistent aggression [5] and several social cognitive and affective factors have been identified as predictors and correlates of aggression. However, to our knowledge, no studies have investigated the joint influences of these factors on forms (relational and physical) and functions (reactive and proactive) of aggression before school age [6]. To address these gaps in the literature, we examined whether two well-implicated emotion factors - empathy and anger - mediated the association between children’s general normative beliefs about aggression (GNBAA) and their actual aggressive behavior.

### Forms and Functions of Aggression

Aggression is defined as the infliction of harm with malicious intent [7, 8]. Two forms of aggression - relational and physical aggression - have received the most empirical attention in the early childhood developmental period. Relational aggression includes behaviors intended to damage peer relationships and social standing through manipulation whereas physical aggression is the intent to hurt, harm or injure using physical force (e.g. hitting) [1, 9]. Although relational and physical aggression are highly correlated, each form of aggression has distinct developmental trajectories, correlates, and prevalence rates across the lifespan [10–12]. During early childhood, physical aggression generally starts to decline from the ages of two to four as children become more cognitively and verbally mature and aware of social expectations and norms [13]. An alternative behavior, relational aggression, substantially increases between the ages of four to seven [14, 15].

Researchers have identified that aggression may serve varied but distinct functions [7, 16, 17] which can be seen across aggression forms. Proactive aggression is deliberate behavior that is used to obtain a desired object, outcome or self-serving goal. Proactive physical and relational aggression has been shown to provide some protective functions and positive outcomes in young and school-age children such as peer acceptance and improved social status [18, 17, 19]. Theoretically, proactively aggressive children may have their behavior reinforced by peers as they become well-liked and achieve greater social status among their peers. These reinforcing factors may contribute to the increase of relational aggression that is typically observed during early childhood. Reactive aggression, on the other hand, is hostile or impulsive behavior used in response to a perceived threat [20, 16]. While reactive aggression tends to be associated with physical aggression during the early years [21], young children have also been observed engaging in reactive relational aggression [22, 17]. Unlike proactive forms of aggression, reactive physical and relational aggression has been linked to negative social and emotional outcomes such as peer rejection, anger, and poor emotion regulation [20]. Research has shown that these functions are related to different social cognitive and affective skills [18, 23]. This study will use a two-dimensional combination approach (proactive relational, reactive relational, proactive physical, and reactive physical) [21, 17] to document the theoretically meaningful distinctions between forms and functions of aggression and the joint influences of social cognitive (normative beliefs) and affective (empathy and anger) factors.

### Aggression and Normative Beliefs

When social cognitive models have been applied to understanding aggression in older children, ample support has been found for the pathway from normative beliefs to actual behavior [24–26]. Normative beliefs are cognitive standards about the acceptability of aggressive behavior [25] and are situation specific (e.g. “If others hit you first, it is OK to hit them back”) or general (e.g. “It is OK to hit others”), and these beliefs set internal parameters that regulate an individual’s personal actions and behaviors. Individuals who hold normative beliefs about aggression, view aggression as acceptable behavior. A longitudinal study of German adolescents by Krahé and Busching [26], for example, found that approval of aggression concurrently and prospectively predicted the corresponding form of aggression. That is, approval of relational aggression predicted adolescents’ current use of relational aggression and use of relational aggression four years later. The same relationship was found for physical aggression but concurrent associations only, suggesting that in adolescence, normative acceptance of physical aggression may decrease quicker compared to acceptance of relational aggression. The belief-behavior pathways demonstrated in this study of adolescents did not differ in boys and girls.

Few studies have considered normative beliefs in younger children, with the exception of Goldstein and colleagues [27] and Swit and colleagues [28]. Goldstein and colleagues [27] found that preschool-age children viewed relational aggression as more normative than physical and verbal aggression, however, they did not include an assessment of children’s actual aggressive behavior. Swit and colleagues [28] found no differences in relationally aggressive and non-aggressive children’s normative beliefs about relational and physical aggression. However, their assessment of the belief-behavior pathway was limited by a small sample size and there was no distinction between forms and functions of aggression. The lack of empirical research on the belief-behavior pathway in early childhood populations may be due to several reasons. First, there are inherent challenges in assessing very young children’s social cognitive processes such as normative beliefs, particularly when methods have relied on verbal delivery of hypothetical vignettes and questioning procedures [28]. Second and most importantly, some researchers have suggested that normative beliefs may be an unreliable predictor of children’s aggressive behavior until the age of eight when a greater awareness of social norms has developed [29, 25, 30]. However, we argue that early in development, children develop working models and knowledge structures that they draw on to make a judgement about the acceptability of different behaviors. Children who are exposed to aggression may come to believe, early on, that these behaviors are acceptable. Thus, normative beliefs should be typically acquired during early childhood and preliminary evidence from Goldstein and colleagues [27] and Swit and colleagues [28] supports this claim.

To our knowledge, only one study has examined the association between forms and functions of aggression and normative beliefs. Bailey and Ostrov [31] found proactive relational aggression and reactive physical aggression were significant predictors of normative beliefs of aggression in a sample of emerging adults. However, due to poor reliability in subscales, a composite normative beliefs score was obtained by combining all aggression types. Also, this study examined the alternative direction of effect with normative beliefs as the outcome variable. Thus, the results of this study do not allow us to draw conclusions regarding the belief-behavior pathway for each of the corresponding forms and functions of aggression (i.e. normative beliefs about relational aggression and relationally aggressive behaviors). Moreover, given the natural maturation that occurs in children’s social cognitive abilities, especially during early childhood, the belief-behavior pathways identified in previous research with older children may differ from that in an early childhood sample.

### Aggression and Affective Processes

The General Aggression Model [7] builds on social cognitive models of aggression by acknowledging the role of affective processes in increasing a person’s likelihood to aggress. For instance, when a child experiences feelings of anger, this may increase the accessibility of pathways to aggressive cognitions and behavior, particularly if the child holds normative beliefs approving of aggression. Alternatively, empathy is an important predictor of prosocial behavior [32] and decreases aggression as the child can understand what the other child may be experiencing and/or feeling [33]. While some researchers acknowledge that relational and physical forms of aggression are highly related [21, 18], other research has revealed that forms and functions of aggression are differentially associated with affective processes such as empathy and anger.

Empathy, defined as recognising and experiencing the feelings and emotions of others [34], is an important socio-emotional process that fosters warm and positive social relationships and is related to less spontaneous aggression as children get older [35, 36]. Empathic behavior generally enables children to demonstrate care and sensitivity toward others by understanding what they feel (cognitive empathy), and seeing the situation from others’ perspectives (affective empathy). Two predominant views have been examined, theoretically and empirically, regarding the pathway between aggression and empathy. First, aggressors have been shown to display social cognitive deficits and low empathy which makes them more prone to aggression. More specifically, these children have been shown to (mis)interpret social cues and attribute hostile intent to unclear social situations while concurrently having difficulties recognising and processing emotional cues in others. Combined deficits in social processing and cognitive and affective empathy may facilitate aggressive responding [37]. The second alternate view is that aggressors are skilled manipulators, who use a combination of prosocial behavior and empathy to achieve their proactive goals [38, 39]. More specifically, relational forms and proactive functions of aggression require the aggressor to have adequate empathy to know what behaviors will more effectively harm or manipulate others. Thus, greater empathy and social cognitive abilities are thought to be differentially associated with proactive aggression and relational aggression [40, 17, 41, 42], challenging the view that aggressors do not always fit the deficit stereotype often associated with aggressive behavior.

Anger, defined as an emotion felt in response to a perceived or actual threat, when activated, serves to warn or intimidate others [43, 44], has long been implicated in reactive, but not proactive aggression [45, 16, 46]. However, the experience of intense anger does not always translate into aggression and thus, may play an important role in the relationship between social cognitive processes and aggressive behavior [47].

The pathway between anger and aggression has been demonstrated in physical and relational forms of aggression as early as the preschool years. Using an observational measure of aggression and teacher reports of anger, Ostrov and colleagues [17] found reactive and proactive forms of aggression to be differentially associated with anger both concurrently and prospectively. Concurrently, reactive and proactive physical aggression and reactive relational aggression were positively associated with increases in anger. Prospectively, anger was significantly associated with increases in reactive and proactive physical aggression and proactive relational aggression across the four-month study. Despite the strong theoretical link between aggression and anger, there remains a paucity of research examining this relationship within a two-dimensional combination approach of forms and functions of aggression during early childhood. Given the preliminary support for the differential pathways between forms and functions of aggression and anger in young children, replication and extension of these findings are needed [17].

Based on the above literature and theory, it could be reasonably inferred that empathic behavior and anger could mediate the association between children’s normative beliefs about aggression and their actual aggressive behavior. More specifically, children who approve of physical aggression may lack emotional sensitivity towards peers and have problems in managing their anger, increasing their use of reactive and proactive physical aggression and reactive relational aggression. In contrast, children who approve of relational aggression may use empathic behaviors, not anger, to manipulate social relationships to achieve their personal goals and motives, increasing their use of proactive relational aggression.

### Developmental Considerations for Early Childhood

The early childhood developmental period is crucial in children’s development of social norms. Children are beginning to develop cognitive understanding and awareness of appropriate social behaviors to use within different contexts. To this end, it is well implicated that children’s aggression can and should be assessed according to forms and functions, however, relatively little is known about the development of these behaviors during early childhood. Moreover, there is still much to understand about the internal cognitive processes involved in young children’s use of forms and functions of aggression and advances still need to be made regarding the use of developmentally appropriate and innovative measures to assess the social cognition of young children.

### The Current Study

**Fig. 1:**
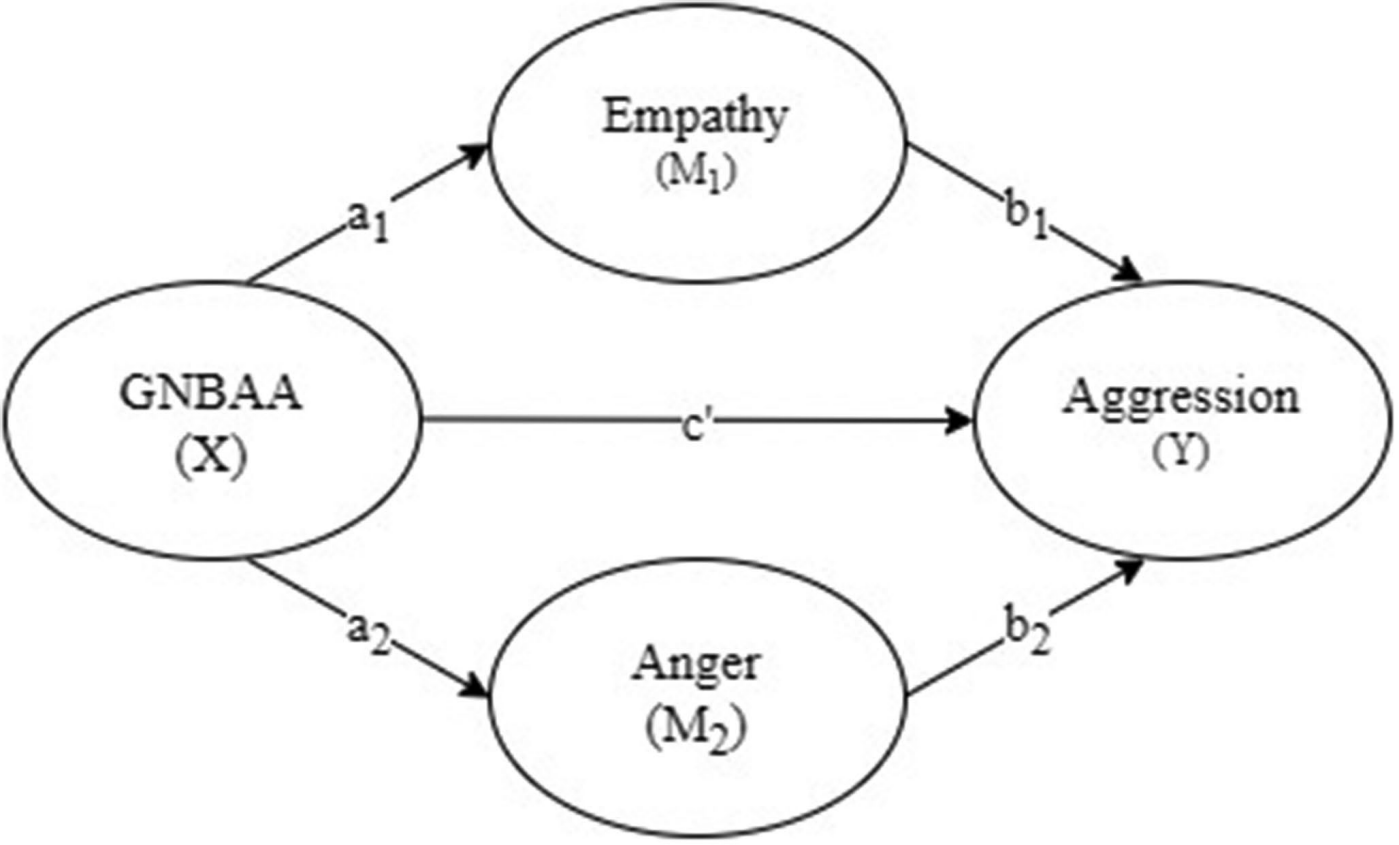
Conceptual diagram examining (1) the indirect effect of GNBAA (X) on Aggression (Y) through Empathy (M_1_) only through a_1_b_1_, (2) the indirect effect of X on Y through Anger (M_2_) only through a_2_b_2_(3) the direct effect of X on Y = c’. Age (C_1_) and Gender (C_2_) were included as covariates for X, M_1_, M_2_, and Y (not displayed).

The present study aims to examine how GNBAA affect the corresponding aggressive behavior in a sample of preschool-age children. As a further step, we will also simultaneously investigate the mediating roles of empathy and anger of children in the association between children’s GNBAA and reactive and proactive functions of the corresponding form of aggression. Namely, whether children who approve of aggression engage in aggressive behavior because of their lower levels of empathy or by their higher levels of anger. To address this, a two mediator model was tested (see Fig. 1) and the following hypotheses were proposed:

#### Hypothesis 1

GNBAA will correlate with low empathy, high anger, and the corresponding form of aggression (i.e. child approval of relational aggression will be associated with teacher-reported reactive and proactive relational aggression).

#### Hypothesis 2

Low empathy (M1) will mediate the direct relation between GNBAA (X) and the corresponding form of aggression (Y); path a1*b1.

#### Hypothesis 3

High anger (M2) will mediate the direct relation between GNBAA (X) and the corresponding form of aggression (Y); path a2*b2.

#### Hypothesis 4

The mediation paths (empathy: a1*b1; anger: a2*b2) will show differentially associated relationships with reactive and proactive functions of aggression.

## Methods

### Participants

A total of 98 children (54.1% males; *M*_*age*_ = 46.21 months, *SD* = 8.84 months) from three community-based kindergartens in three urban, moderate-sized communities in the South Island of New Zealand participated in this study. The age range for the sample was 25–65 months and all children enrolled at the preschools were eligible to participate. School deciles measure the socio-economic position of a school’s student community compared to other schools throughout the country. Scores range from one to ten, with a lower score indicating a higher proportion of students from low socio-economic communities and a higher score representing fewer of these students [48]. The Kindergartens were located in decile three, five and eight communities suggesting a diverse sample of socioeconomic status. Participation rates at all Kindergartens exceeded 80%. The sample was composed of the following ethnic groups: 65% Caucasian, 15% Māori, 6% Pacific Islander, and the remaining 10% from Southeast Asia and European countries.

Ten kindergarten teachers (87.5% female, *M*_*age*_ = 57.70 years, *SD* = 3.61) completed teacher reports of children’s reactive and proactive relational aggression, reactive and proactive physical aggression, empathy, and anger. All teachers identified as Caucasian. Six teachers had completed a Bachelors degree; four had completed a Diploma. Teachers had nine to thirty years (*M* = 23.3 years, *SD* = 7.40) experience working in Kindergartens.

### Measures

#### Teacher Report of Forms and Functions of Aggression

The Preschool Proactive and Reactive Aggression – Teacher Report (PPRA-TR; [49]) was used to assess proactive relational aggression (3 items; e.g., “This child often says “you can’t come to my birthday party” to other children to get what s/he wants”); reactive relational aggression (3 items; e.g., “When s/he is upset with others, this child will often ignore or stop talking to them”); proactive physical aggression (3 items; e.g., “This child often starts physical fights to get what s/he wants”); reactive physical aggression (3 items; e.g., “If other children anger this child, s/he will often hit, kick, or punch them”); and two positively toned filler items. Teachers responded on a 5-point Likert-type scale from 0 (never or almost never) to 4 (always or almost always). Scores were summed and averaged for each subscale. Cronbach’s alpha for this study was 0.87 for reactive relational aggression, 0.91 for proactive relational aggression, 0.94 for reactive physical aggression, and 0.85 for proactive physical aggression.

The National Institute of Health Toolbox Emotion Battery (NIHTB-EB) surveys were developed as parent reports. The items assessing empathic concern and anger were adapted to read “this child” and have been used as teacher reports for this study. Each survey demonstrated good internal consistency, suggesting that teacher informants can reliably report on these constructs using the NIHTB-EB surveys.

#### Teacher Report of Children’s Empathic Behavior

The NIHTB-EB Empathic Behavior Ages 3–12 v2.0 [50] consists of ten items (e.g. “This child tries to help someone who has been hurt”) assessing how often a child shows empathic behaviors towards peers. Empathic concern was measured on a 5-point Likert-type scale from 0 (Never) to 4 (Always). Higher scores are indicative of more teacher-reported empathic behaviors. Reliability was excellent, with Cronbach’s α equal to 0.94.

#### Teacher Report of Children’s Anger

The NIHTB-EB Anger Ages 3–12 v2.0 [50] consists of nine items (e.g. “This child argues a lot with adults”) assessing how often a child displays an angry mood towards peers and adults. Teachers were asked to report on a child’s anger measured on a 3-point Likert-type scale from 0 (Never or not true) to 2 (Often or very true) with higher scores indicating more child anger. For this study, Cronbach’s α was 0.85.

#### Normative Beliefs Towards Relational Aggression and Physical Aggression

An assessment of children’s normative beliefs about relational and physical aggression was conducted using an adapted interview based on prior research in early childhood [28]. The interview consisted of three vignettes that were enacted using figurines and animations shown on a tablet. A description of the vignettes can be found in the Supplementary Material. Each of the vignettes lasted between 31 and 38 s. To reduce the likelihood of gender biases and responses influenced by emotionality, the vignettes were designed to be gender-neutral and no expressions were shown by the characters. The interview with children was developed by the first author who had previous experience using similar toy- and screen-based measures to assess young children’s social cognitive processes (e.g., theory of mind tasks).

**Fig. 2:**
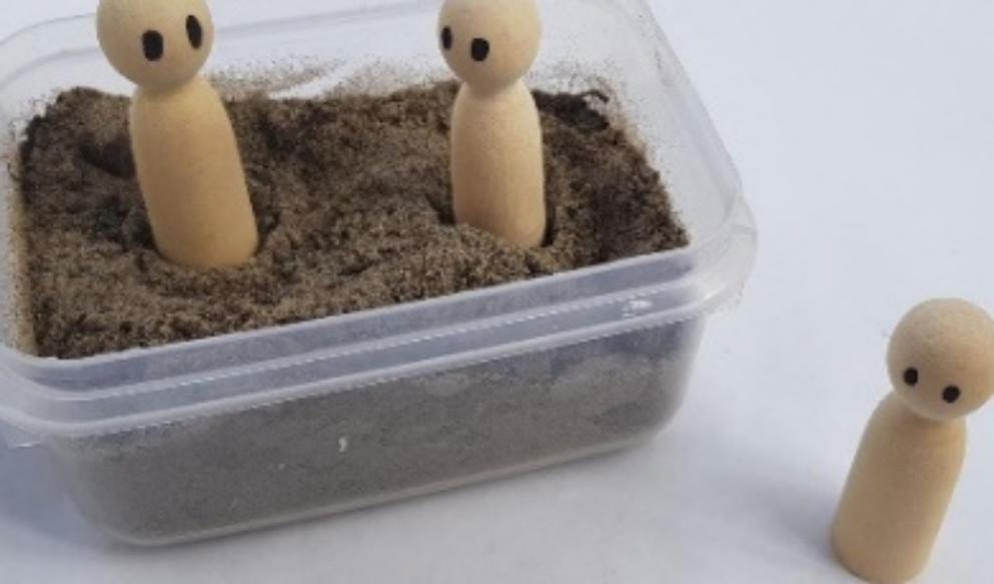
Props used to Describe Hypothetical Vignettes

**Fig. 3:**
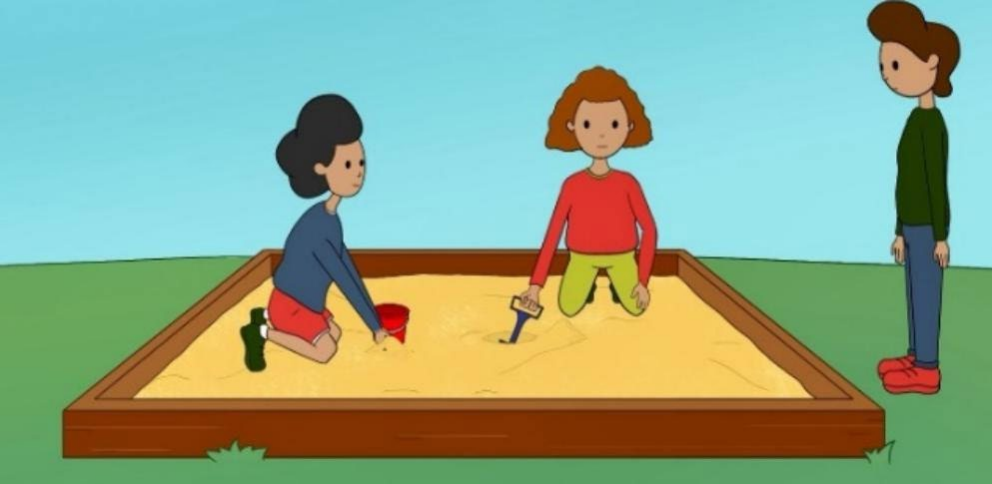
Animations used to Describe Hypothetical Vignettes

The researcher first described the vignette. For example, “these children are playing in the sandpit. Another child walks over to play. This child says ‘you can’t play with us, go away.’” The accompanying props include a container with sand and three gender-neutral figurines (see Fig. 2). The animation of the vignette shown to children on the tablet parallels the scenario using figurines (see Fig. 3). Children’s responses to the vignettes were recorded for later coding.

Following each vignette, children were asked a series of questions designed to assess (1) their beliefs about the acceptability of different types of aggressive provocation (relational and physical), and (2) the behavioral responses they thought would follow after the aggression. These questions were adapted from previous research by Huesmann and Guerra [25] and Swit and colleagues [28]. The assessment of normative beliefs is the focus of this paper.

**Fig. 4:**
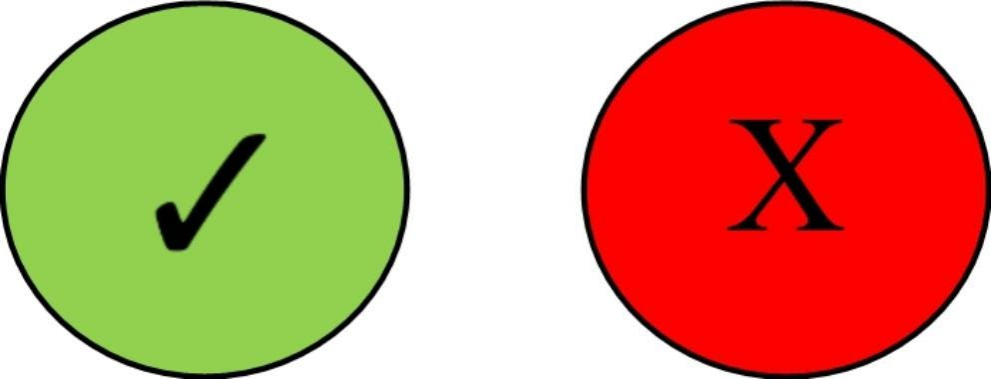
Normative Beliefs Response Options

Beliefs about the acceptability of aggression were assessed by asking children, “Is it okay to [say ‘you can’t play with us, go away’]?” with response options of “yes” or “no.” Children indicated their response by pointing to a cross or a tick (see Fig. 4). If the child indicated “yes”, they were asked if the scenario was “a little bit okay” or “very okay”. If the response was “no”, the child was asked if the scenario was “a little bit wrong” or “very wrong”. Children’s verbal responses were supported by two circles; a small circle representing “a little bit okay/wrong” and a large circle representing ‘’very okay/wrong”. Beliefs were coded numerically (1 = Aggression is very wrong, 2 = Aggression is a little bit wrong, 3 = Aggression is a little bit okay, 4 = Aggression is very okay). Similar scales have been used previously to measure acceptability beliefs in preschool- (e.g., [28]) and school-age children (e.g., [25]). However, no studies have employed two different modes of enactment of vignettes using a counterbalanced design. Children’s normative beliefs were assessed twice, on two separate days. A total composite normative beliefs score (ranging from 1 to 4) was obtained for relational aggression and physical aggression by summing and averaging children’s scores from both sessions. Higher scores reflected greater approval of the behavior. Children’s normative belief scores for each interview session were highly correlated, indicating adequate test-retest reliability (*r* = 0.44–0.70) across the two counterbalanced interview sessions.

### Procedures

The study was approved by the University’s Human Research Ethics committee, and parents provided written informed consent before child participation. Child assent was obtained before completing the GNBAA interviews. Children participated in two alternate designs of the interview on two separate days, no more than one-week apart. Children were randomly allocated to a design in session one. In session two, children completed an alternate design. To assess normative beliefs, there were four counterbalanced conditions: 2 (order of vignette) x 2 (order of modality). This means that within each child, the order of administration of the vignettes was counterbalanced with prosocial behavior being presented as the second vignette in one of the sessions, and the order of enacting the vignette using figurines or animation was also counterbalanced across children. More specifically, over the two sessions, children responded to both animation and figurine scenarios for each normative belief. The prosocial behavior normative belief was not included in this analysis because it was not of interest to this research question. On average, the administration of all three vignettes took 4.22 min. Ninety-three percent of children (*n* = 62) completed both sessions. The interviews took place in a quiet area in the kindergarten, away from distractions. After each session, children received a sticker of their choice. The full measure including the animations is available upon request from the first author. Teachers provided written informed consent before completing the behavioral reports. Teacher reports were distributed and completed two weeks before the completion of data collection. Teachers who had known the child for at least 8 weeks completed the behavioral reports.

### Data Analysis

All the data analyses were conducted with SPSS 28.0 software package. First, analyses were conducted to examine patterns of missing data. Teacher reports of children’s aggression, empathy and anger were complete. Nine children (9%) did not complete the second normative beliefs interview. These children’s scores from the first normative beliefs interview were used in the analysis. Next, data reliability estimates, assessment of normality, and descriptive statistics were examined. Outliers were defined as any value greater than 3SD above or below the mean. The Winsorization procedure was used and outliers were modified to +/-3SD of the mean [51]. Pearson’s correlation was used to examine the relationships among key study variables. The hypothesised mediation models for each form and function of aggression (Fig. 1) were tested using the PROCESS macro in SPSS (Model 4) developed by Hayes [52] http://www.afhayes.com. Bias-corrected bootstap confidence intervals (CIs) derived from 5000 boostrap resamples are estimated to test the indirect effects. The indirect effect is considered significant if zero is not included in the CIs [52]. Age and gender were controlled for in the statistical analyses due to their associations with child aggression (*r* = 0.20 − 0.22 for this study). We exclude covariates from display in Fig. 1 for ease of interpretation.

## Results

### Preliminary Analyses

Descriptive statistics and correlations between key study variables are presented in Table 1 (for gender differences in key study variables see Supplemental Materials). The results showed GNBAA were not associated with the corresponding form of aggression. Both GNBAA relational aggression and GNBAA physical aggression were significantly and negatively associated with empathy but not anger. All four forms and functions of aggression were positively associated with anger and these relations were moderate to strong. Proactive relational aggression was significantly positively associated with empathy, whereas proactive physical aggression was negatively associated with empathy. Reactive relational aggression and reactive physical aggression were not significantly associated with empathy. Significant associations across measures of aggression were found, with the strongest associations existing within aggression forms.

**Table 1:** Descriptive Statistics, and Correlations of GNBAA, Empathy, Anger, and Forms and Functions of Aggression

	1	2	3	4	5	6	7	8
1. GNBAA RA	-							
2. GNBAA PA	0.53***	-						
3. Empathy	− 0.26**	− 0.22*	-					
4. Anger	− 0.02	− 0.15	− 0.26**	-				
5. R-RA	− 0.06	− 0.19	0.18	0.53***	-			
6. P-RA	− 0.00	− 0.18	0.20*	0.40***	0.85***	-		
7. R-PA	0.08	− 0.09	− 0.19	0.73***	0.37***	0.28**	-	
8. P-PA	0.13	− 0.00	− 0.25**	0.70***	0.19	0.14	0.81***	-
Mean (SD)	1.78 (0.94)	1.71 (0.92)	2.18 (0.92)	0.45 (0.50)	1.00 (0.99)	0.59 (0.86)	0.66 (0.87)	0.26 (0.59)
Range	1–4	1–4	0-3.70	0–2	0–3	0–3	0-3.27	0-2.18

⁎ *p* < 0.05. ⁎⁎ *p* < 0.01. ⁎⁎⁎ *p* < 0.001. GNBAA = General Normative Beliefs about Aggression; R-RA = Reactive Relational Aggression; P-RA = Proactive Relational Aggression; R-PA = Reactive Physical Aggression; P-PA = Proactive Physical Aggression

### Analyses of the Mediating Roles of Empathy and Anger

*Direct effects.* Results provided partial support for hypothesis 1, showing that children who perceived relational aggression as acceptable behavior, had lower teacher-reported empathic behaviors (path a1) but not anger (path a2) and this finding was consistent for reactive and proactive relational aggression. Conversely, children’s GNBAA physical aggression was not associated with teacher-reported empathy or anger for reactive and proactive physical aggression. The total effect of children’s GNBAA relational aggression in predicting reactive relational aggression was weak, negative, and not statistically significant (path c; B = − 0.01, SE = 0.12, *t* = − 0.06, *p* = 0.95) and the prediction of proactive relational aggression was weak, positive, and not statistically significant (B = 0.05, SE = 0.10, *t* = − 0.51, *p* = 0.61). The total effect of children’s GNBAA physical aggression in predicting reactive physical aggression (B = − 0.08, SE = 0.10, *t* = − 0.78, *p* = 0.44) and proactive physical aggression (B = − 0.00, SE = 0.07, *t* = − 0.03, *p* = 0.97) was weak, negative, and not statistically significant.

*Indirect and total effects.* To test Hypotheses 2, 3 and 4 with regard to relational aggression, we inspected unstandardized estimates and their corresponding standard error terms and bootstrapped confidence intervals to identify the statistically significant indirect effects. The specific indirect path in which empathy was included as the sole mediating variable (a1*b1) was statistically significant in predicting reactive relational aggression and proactive relational aggression (R-RA: IE = − 0.08, 95%CI [-0.16, − 0.00]; P-RA: IE = − 0.07, 95%CI= [-0.15, − 0.00]). The indirect effect of anger as the mediating variable (a2*b2) was not significant in predicting reactive relational aggression (IE = 0.04, 95%CI [-0.10, 0.17]) or proactive relational aggression (IE = − 0.03, 95%CI= [-0.07, 0.12]). We concluded that empathy has the potential to mediate the effect between children’s GNBAA relational aggression and reactive relational aggression (total effect = − 0.01 and direct effect = 0.03) and proactive relational aggression (total effect = − 0.05 and direct effect = 0.09). We found noteworthy trends regarding the two covariates, gender and age. As shown in Tables 2 and 3, approval of relational aggression was higher among younger children. Moreover, teachers reported that girls were more likely to use reactive relational aggression and proactive relational aggression compared to boys.

**Table 2:** Model Testing the Two Mediator Model with Reactive Relational Aggression

Predictors	X (GNBAA)		M1 (Empathy)		M2 (Anger)		Y (Aggression)	
	β	t	β	t	β	t	β	t
X (GNBAA)	-	-	− 0.22	− 2.06*	− 0.03	0.52	0.03	0.33
M1 (Empathy)	-	-	-	-	-	-	0.35	3.70***
M2 (Anger)	-	-	-	-	-	-	1.23	7.37***
C1 (Gender)	0.09	0.96	0.27	1.49	− 0.08	− 0.78	0.36	2.23*
C2 (Age)	− 0.37	− 3.86***	0.01	0.87	1.47	0.14	− 0.00	− 0.11
F	7.80		3.04		0.88		12.92	
R^2^	0.14***		0.09*		0.03		0.42***	

*N* = 98 ⁎ *p* < 0.05. ⁎⁎ *p* < 0.01. ⁎⁎⁎ *p* < 0.001. Gender: Girls = 1

**Table 3:** Model Testing the Two Mediator Model with Proactive Relational Aggression

Predictors	X (GNBAA)		M1 (Empathy)		M2 (Anger)		Y (Aggression)	
	β	t	β	t	β	t	β	t
X (GNBAA)	-	-	− 0.22	− 2.06*	0.03	0.52	0.09	1.02
M1 (Empathy)	-	-	-	-	-	-	0.30	3.36***
M2 (Anger)	-	-	-	-	-	-	0.85	5.35***
C1 (Gender)	0.09	0.96	0.27	1.49	− 0.08	− 0.78	0.32	2.07*
C2 (Age)	− 0.37	− 3.86***	0.01	0.87	0.00	1.47	0.00	0.43
F	7.80		3.04		0.88		8.00	
R^2^	0.14***		0.09*		0.03		0.31***	

*N* = 98 ⁎ *p* < 0.05. ⁎⁎ *p* < 0.01. ⁎⁎⁎ *p* < 0.001. Gender: Girls = 1

Next, we examined hypotheses 2, 3, and 4 with regard to physical aggression. The specific indirect path in which empathy was included as the sole mediating variable (a1*b1) was not statistically significant in predicting reactive physical aggression and proactive physical aggression (R-PA: IE = − 0.00, 95%CI= [-0.04, 0.03]; P-PA: IE = − 0.00, 95%CI= [-0.02, 0.02]). The indirect effect of anger as the mediating variable (a2*b2) was not significant in predicting reactive physical aggression (IE = 0.04, 95%CI [-0.10, 0.17]) or proactive physical aggression (IE = − 0.05, 95%CI= [-0.14, 0.04]). We concluded that neither empathy nor anger influences the potential relationship between children’s GNBAA physical aggression and reactive and proactive physical aggression.

However, it is noteworthy that the relationship direction between children’s GNBAA physical aggression and reactive and proactive physical aggression changed when both mediators were added to the model (R-PA total effect = − 0.08 and direct effect = 0.00; P-PA total effect = − 0.00 and direct effect = 0.05) suggesting that while the mediating effect did not reach significance in this study, empathy and anger do influence the relationship between GNBAA and physical aggression. With regards to the covariates (see Tables 4 and 5), approval of physical aggression was higher among younger children. Moreover, teachers reported that boys were more likely to use reactive physical aggression compared to girls.

**Table 4:** Model Testing the Two Mediator Model with Reactive Physical Aggression

Predictors	X (GNBAA)		M1 (Empathy)		M2 (Anger)		Y (Aggression)	
	β	t	β	t	β	t	β	T
X (GNBAA)	-	-	− 0.18	-1.60	− 0.06	− 0.99	0.00	0.04
M1 (Empathy)	-	-	-	-	-	-	0.04	0.55
M2 (Anger)	-	-	-	-	-	-	1.28	10.10***
C1 (Gender)	0.04	0.43	0.25	1.35	− 0.07	− 0.69	− 0.30	− 2.52**
C2 (Age)	− 0.36	− 3.70***	0.01	1.05	0.01	0.94	− 0.01	− 0.96
F	6.90**		2.45		1.13		24.42	
R^2^	0.13		0.07		0.04		0.57***	

*N* = 98 ⁎ *p* < 0.05. ⁎⁎ *p* < 0.01. ⁎⁎⁎ *p* < 0.001. Gender: Girls = 1

**Table 5:** Model Testing the Two Mediator Model with Proactive Physical Aggression

Predictors	X (GNBAA)		M1 (Empathy)		M2 (Anger)		Y (Aggression)	
	β	t	β	t	β	t	β	t
X (GNBAA)	-	-	− 0.18	− 1.60	− 0.06	− 0.99	0.05	0.90
M1 (Empathy)	-	-	-	-	-	-	− 0.01	− 0.20
M2 (Anger)	-	-	-	-	-	-	0.83	9.24***
C1 (Gender)	0.04	0.43	0.25	1.35	− 0.07	− 0.69	− 0.15	-1.74
C2 (Age)	− 0.36	− 3.70***	0.01	1.05	0.01	− 0.94	− 0.01	− 1.50
F	6.90**		2.45		1.13		20.60	
R^2^	0.128		0.07		0.04		0.53***	

*N* = 98 ⁎ *p* < 0.05. ⁎⁎ *p* < 0.01. ⁎⁎⁎ *p* < 0.001. Gender: Girls = 1

## Discussion

This study implemented a two-mediator model to examine the influence of empathy and anger on the relationship between GNBAA and child aggression. A two-dimensional model of aggression was used whereby forms and functions of aggression were examined in an early childhood sample using teacher reports. Results generated several new insights into the role of social cognitive and affective factors on early childhood aggressive behavior.

Firstly, no association between GNBAA and the corresponding form of aggression was identified (Hypothesis 1). The current findings differ from those found in previous research with older children (e.g [26]. These discrepancies in findings suggest that GNBAA, while present, may not be sufficient in predicting aggressive behavior during the early childhood developmental period. While normative beliefs about the acceptability of aggressive behavior are stable cognitive constructs, the pioneering work of Huesmann & Guerra [25] established that normative beliefs, which develop as a function of social experiences and cognitive growth, were unstable in early childhood relative to middle childhood and adolescence. Moreover, according to the General Aggression Model [7], when scripts approving of aggression are frequently rehearsed and activated, they become chronically accessible and aggressive behavior becomes automatic [53]. Thus, the belief-behavior pathway that is frequently found in school-age children may be the result of greater and more frequent opportunities for rehearsal of aggressive scripts compared to young children.

Across aggression forms, reactive aggression was found to be the most frequently expressed function in this early childhood sample. Relative to proactive aggression, reactive functions of relational and physical aggression are conceptualized as resulting from the experience of frustration whereas proactive aggression is mediated by social learning processes and operant learning principles [54]. As such, it is likely that both the development and maturation of social cognition and the high prevalence of reactive aggression (the function of aggression that is less likely to recruit social cognitions and beliefs) contributed to the lack of effect between GNBAA and children’s aggressive behavior.

Interestingly, GNBAA were not associated with anger (Hypothesis 1), but not surprisingly, anger was positively associated with all forms and functions of aggression. Anger is a frequently occurring emotion across development and is at least partially a function of bottom-up, non-cognitively mediated, processes [55], which may explain the lack of association between GNBAA, anger and forms and functions of aggression (Hypothesis 3 & 4). Associations were most robust for measures of physical aggression and, across functions, stronger associations were seen on measures of reactive aggression. These results indicate that angry emotions are powerful factors contributing to aggressive behavior in early childhood.

Unlike anger, empathy was negatively associated with GNBAA relational aggression and physical aggression (Hypothesis 1). Mediational analyses revealed significant associations between GNBAA, empathy, and proactive and reactive forms of relational, but not physical aggression (Hypothesis 2 & 4). Associations were such that for both reactive and proactive relational aggression, endorsement of the acceptability of aggression was positively associated with empathy. Interestingly, higher empathy was associated with increased use of relational aggression and less use of physical aggression. These mixed associations can be understood from a developmental perspective.

Empathy is a cognitive process that requires perspective-taking and is also an emotion-based reaction [56]. The capacity to understand emotions in others informs social-cognitive development and has been shown to develop across childhood [57]. Regarding the negative association between GNBAA relational aggression, GNBAA physical aggression, and empathy, it may be that these developing social-emotional processes protect against the co-occurring development and maintenance of social cognitions such as normative beliefs accepting of relational aggression.

However, as children develop, learn about and interact in social situations, there are additional opportunities to aggress. Relational aggression, behaviors intended to damage peer relationships and social standing, onsets as a function of growing awareness of social relationships. In contrast to early emerging physical aggression, past studies have shown that while relational aggression occurs in early childhood, these behaviors are developing in parallel with increasing social understanding and cognitive growth, and, as such, are rudimentary, and often direct behaviors rather than nuanced or indirect [58]. The positive association identified between empathy and relational aggression likely reflects this development. Further, as there was no direct effect identified between GNBAA and teacher-reported relational aggression, it appears that the relationship between social-emotional awareness and relational aggression exists in the absence of cognitions promoting the acceptability of such behaviors. As such, based on the dynamic growth in social and cognitive/emotional processes during early childhood, it is likely that the positive association seen between empathy and both reactive and proactive relational aggression is a reflection of inchoate processes associated with dynamic changes as part of early childhood development.

Our results provide initial evidence that GNBAA are changing as a function of age. More specifically, young children perceived relational and physical aggression as more acceptable compared to older children. While this is what we would developmentally expect of children’s GNBAA, more research is needed to determine when GNBAA becomes a robust predictor of child aggression. The results of the present study also provide additional support for the distinctive differences in the development of forms (relational and physical) and functions (reactive and proactive) of aggression in early childhood in a cultural context outside of the United States and Europe.

### Limitations and Future Directions

There are many strengths of the present study including the focus on social cognitive (GNBAA) and affective factors in a diverse early childhood sample. The use of a counterbalanced study design using iconic (animation) and enactive (toy figurines) hypothetical scenarios appeared to be an acceptable and developmentally appropriate approach to studying young children’s GNBAA. Despite these strengths, there are limitations to this study. The cross-sectional nature of our study limited the ability to infer the direction of causality. Theoretically and empirically, ongoing use of aggression may influence and strengthen children’s GNBAA. Future work using a longitudinal design or experimental methods is needed to ascertain the direction of causality. Moreover, we were only able to examine these associations based on teachers’ behavioral reports of child aggression, empathy and anger. Future research should expand upon this work by using other informants such as peer reports and observational methods. This study only focused on empathic concern, however, cognitive empathy and affective empathy have been shown to perform different roles in relation to aggressive behavior [59]. Thus, future research should examine cognitive and affective empathy in the mediation of the relationship between GNBAA and child aggression. Despite these limitations, the findings of this study add to our understanding of the distinction between subtypes of aggressive behavior in young children and the social cognitive (general normative beliefs) and affective (empathy and anger) processes that influence these behaviors.

### Summary

In this study, we were interested in understanding the mediating effects of empathy and anger in the association between children’s GNBAA and forms (relational and physical) and functions (reactive and proactive) of aggressive behavior. We hypothesised that (1) GNBAA will correlate with low empathy, high anger, and the corresponding form of aggression; (2) low empathy will mediate the relation between GNBAA and the corresponding form of aggression; (3) high anger will mediate the relation between GNBAA and the corresponding form of aggression; and (4) both mediation paths will be differentially associated with reactive and proactive functions of aggression. We found partial support for our hypotheses. No main effects of GNBAA on the corresponding aggressive behavior were found. Empathy, but not anger, mediated the effect between children’s GNBAA about relational aggression and teacher-reported reactive and proactive relational aggression. Unexpectedly, neither empathy nor anger influenced the relationship between children’s GNBAA about physical aggression and teacher-reported reactive and proactive physical aggression.

## Electronic Supplementary Material

Below is the link to the electronic supplementary material.

Supplementary Material 1

## Data Availability

The data that support the findings of this study are available from the corresponding author, Cara S Swit, upon reasonable request.
