# Supplementary material for: Normative Beliefs and Aggression: The Mediating Roles of Empathy and Anger
Source: Child Psychiatry Hum Dev. 2023 Jun 22;56(1):236–48. doi: 10.1007/s10578-023-01558-1 (PMC11828841; doi:10.1007/s10578-023-01558-1)
Supplement: Supplementary file 1 — Supplementary Material 1 [file 10578_2023_1558_MOESM1_ESM.docx]

Supplementary Material

Table 1. Aggression and Prosocial Behavior Scenarios

| Relational Aggression | These children are playing in the sandpit. Another child walks over to play. The child says “you can’t play with us, go away.” |
| --- | --- |
| Physical Aggression | These children are playing with the blocks. This child throws a block at the other child. |
| Prosocial Behavior | This child is colouring in. Another child sits down to colour in as well. The child gives the child a crayon to use. |

Table 2. Gender Differences for all Main Study Variables and ANOVA results

|  | Boys | | Girls | | t | *p* |
| --- | --- | --- | --- | --- | --- | --- |
|  | M | SD | M | SD |  |  |
| GNBAA RA | 1.73 | 0.96 | 1.83 | 0.93 | -.56 | .58 |
| GNBAA PA | 1.70 | 0.93 | 1.72 | 0.91 | -.13 | .90 |
| Empathy | 2.06 | 0.98 | 2.32 | 0.85 | -1.39 | .17 |
| Anger | 0.48 | 0.53 | 0.42 | 0.47 | .65 | .52 |
| R-RA | 0.82 | 0.99 | 1.20 | 0.96 | -1.90 | .06† |
| P-RA | 0.42 | 0.74 | 0.78 | 0.96 | -2.06 | .04* |
| R-PA | 0.84 | 0.94 | 0.45 | 0.74 | 2.24 | .03* |
| P-PA | 0.36 | 0.68 | 0.15 | 0.44 | 1.74 | .09† |

† p < 0.10 *p < 0.05 **p < 0.01 *** p < 0.001
